# Assessment of Oral Hygiene Practices, Adjunctive Cleaning Methods, and Their Impact on Plaque and Gingival Indices Among Young Adults in Urban and Rural Settings in Romania

**DOI:** 10.3390/healthcare13222970

**Published:** 2025-11-19

**Authors:** Ioana Elena Lile, Șerban Talpoș-Niculescu, Diana Marian, Otilia Stana, Andra-Alexandra Stăncioiu, Alexandru Cătălin Motofelea, George-Dumitru Constantin

**Affiliations:** 1 Department of Dentistry, Faculty of Dentistry, “Vasile Goldiș” Western University of Arad, 94-96 Revolutiei Blvd., 310025 Arad, Romania; lile.ioana@uvvg.ro (I.E.L.); stancioiu.andra@uvvg.ro (A.-A.S.); 2 Discipline of Oral and Maxillo-Facial Surgery, Faculty of Dental Medicine, “Victor Babes” University of Medicine and Pharmacy, 300062 Timișoara, Romania; talpos.serban@umft.ro; 3 Center for Molecular Research in Nephrology and Vascular Disease, Discipline of Nephrology, Department VII/Internal Medicine II, Faculty of Medicine, “Victor Babeș” University of Medicine and Pharmacy, 300041 Timișoara, Romania; alexandru.motofelea@umft.ro; 4 Discipline of Clinical Practical Skills, Department I Nursing, Faculty of Medicine, “Victor Babeș” University of Medicine and Pharmacy, 300041 Timișoara, Romania; george.constantin@umft.ro

**Keywords:** activated charcoal, adjunctive cleaning, bass technique, dental plaque, gingivitis, lemon, oral hygiene, periodontal health, sodium bicarbonate, toothbrushing frequency, urban–rural differences, young adults

## Abstract

Background/Objectives: Young adults increasingly prefer natural dentifrices; however, the short-term clinical effects and behavioural correlates of these products remain insufficiently documented. This study aimed to evaluate the associations between daily oral-hygiene practices, adjunctive cleaning methods, and plaque and gingival indices, and to compare the short-term effects of three natural toothpaste formulations—sodium bicarbonate, activated charcoal, and lemon-based. Methods: In this cross-sectional study, 101 Romanian adults (aged 20–41 years; 69.3% urban) completed a structured questionnaire assessing brushing frequency and the use of dental floss, interdental stimulators, and mouth rinses. Clinical assessments included the Silness–Löe Plaque Index and the Löe–Silness Gingival Index (modified by Lobene), recorded immediately before and after a supervised 2 min brushing session using the Bass technique with soft-bristled brushes and the participant’s self-reported natural toothpaste (baking soda: *n* = 42; charcoal: *n* = 27; lemon: *n* = 32). Results: Before brushing, 78.2% of participants presented with thin adherent plaque, while the gingival status was normal in 72.3%, mildly inflamed in 22.8%, and moderately inflamed in 5.0%. After brushing, the proportion with normal gingiva increased to 77.2%, and the proportion of thin, adherent plaque decreased to 22.8%. Brushing frequency was positively correlated with plaque reduction (ρ = 0.42, *p* < 0.001), while the use of adjunctive cleaning methods was inversely correlated with post-brushing gingival inflammation (ρ = −0.36, *p* = 0.002). Gingival improvement differed significantly among toothpaste types (χ^2^, *p* < 0.001), with formulations containing baking soda and lemon showing slightly more favourable short-term gingival categories than charcoal; these patterns are descriptive and do not demonstrate comparative product efficacy. Urban–rural differences were observed for brushing frequency (*p* < 0.001) and periodontal status with fewer lesions among urban participants (*p* = 0.002). Conclusions: A single supervised Bass-technique brushing session resulted in measurable short-term improvements in plaque and gingival indices among young adults. Sodium bicarbonate and lemon-based dentifrices appeared to be associated with slightly more favourable short-term gingival responses than charcoal, although these findings should be interpreted as preliminary. These findings emphasise the importance of consistent brushing and interdental cleaning behaviours and highlight the need for longitudinal randomised trials to evaluate the durability, safety, and comparative effectiveness of natural versus conventional dentifrices. Given the cross-sectional, single-session design, these findings are exploratory and do not establish causal or comparative product efficacy.

## 1. Introduction

A proteinaceous layer known as the acquired enamel pellicle immediately develops on the enamel surfaces inside the oral cavity. The enamel pellicle is crucial for both healthy and diseased organisms. It safeguards against abrasive harm, serves as a barrier and cushion against mineral-extracting agents, retains electrolytes for remineralization, and eradicates microorganisms [1].

Planktonic bacteria in the oral fluids will bind to specific proteins in the enamel pellicle, hence diminishing the beneficial effects of the pellicle during dental plaque formation [1]. The parotid gland primarily secretes α-amylase, the predominant enzyme in human saliva. The released soluble amylase degrades starch. Amylase, in conjunction with other proteins present in saliva, may associate with the high-molecular-weight mucin MG1. These structures may serve as reservoirs for pellicle precursors. This does not necessarily imply that amylase is not a precursor to pellicle formation [2].

The oral cavity hosts a complex microbial ecosystem dominated by bacterial species, which play a central role in the formation of plaque biofilms and gingival inflammation [3].

Dental plaque consists of microorganisms residing in a biofilm atop a polymer matrix derived from both bacteria and the host organism. Biofilms provide a greater challenge for antimicrobials to eradicate; however, microbial populations may exhibit increased pathogenic synergy. This knowledge is crucial for clinical application. Antimicrobial agents may be unable to penetrate the architecture of the plaque biofilm. Conversely, bacteria that proliferate on a surface exhibit slower growth and possess a distinct phenotype, rendering them less susceptible to inhibitors. Plaque is a natural component that aids in the development of the body’s defences and metabolism, similar to the microflora present in several other regions of the body [4].

Dental plaque develops in a sequential, organised manner of microbial colonisation on the tooth surface [5].

Plaque biofilm develops through successive bacterial colonisation and co-adhesion processes, forming a mature community that is difficult to remove mechanically or chemically [6,7,8].

Currently, dental care is seen as both a social and physical competency for the majority of individuals. Maintaining dental hygiene requires significant financial investment in products such as toothpaste, toothbrushes, mouth rinses, and other essentials. Teeth discolouration is mainly attributed to the use of certain substances, such as coffee, tea, wines, and alcoholic beverages. Regular consumption of some fruits and vegetables, such as potatoes and beets, may alter the colour of your teeth. Regular use of beverages such as coffee, soda, and tannin-rich wines can degrade enamel and lead to tooth discolouration. The discoloration results from the accumulation of nicotine in the enamel due to habitual smoking and tobacco use [9]. Dentists often use professional teeth whitening techniques using bleaching chemicals derived from peroxides (such as hydrogen peroxide, carbamide peroxide, or analogous compounds) in the form of gels, pastes, or rinses. Laser light is sometimes used to activate these compounds. Although these compounds are effective, they may damage enamel, hence increasing safety concerns. Consequently, an increasing number of individuals are exploring natural methods for teeth whitening, such as using salt, fruits, and botanical extracts, which are said to be more effective and economical [9].

Several randomised and clinical trials have evaluated sodium bicarbonate–based dentifrices, demonstrating significantly greater plaque removal and reductions in gingival bleeding compared with non-bicarbonate formulations in adult populations [10]. Recent systematic reviews also highlight that evidence for charcoal-based and lemon/citrus-based dentifrices remains limited and inconsistent, particularly in young adult and rural populations [11]. Therefore, the present study aimed to address these gaps by comparing the short-term effects of three natural formulations among Romanian young adults from both urban and rural settings.

Although similar topics have been explored, few studies have compared several natural toothpastes under standardised brushing conditions or examined differences between urban and rural young adults.

Therefore, the present study aimed to assess the associations between everyday oral-hygiene practices, adjunctive cleaning methods, and plaque/gingival indices, and to compare the short-term effects of three natural dentifrices—sodium bicarbonate, activated charcoal, and lemon—among young adults from urban and rural settings in Romania.

Study Hypotheses

Based on previous literature and the study objectives, the following hypotheses were formulated:

**H1.**  *A single supervised Bass brushing session would result in measurable short-term reductions in plaque and gingival indices across all participants.*

**H2.**  *Participants habitually using sodium bicarbonate and lemon-based dentifrices would exhibit greater immediate improvements in gingival status compared with those using charcoal-based formulations.*

**H3.**  *Higher brushing frequency and the use of adjunctive cleaning methods (floss, interdental stimulators, mouthrinse) would be associated with lower post-brushing plaque and gingival scores.*

**H4.**  *Urban participants would exhibit more favourable oral hygiene behaviours and gingival health indicators than rural participants.*

## 2. Materials and Methods

This study adhered to the guidelines for researching human subjects and was approved by the Institutional Ethics Committee of Vasile Goldiș Western University of Arad (Approval No. 18/02.10.25). After fully understanding the study’s goals, methods, potential benefits, and risks, all participants gave their written permission. We informed the participants that they could withdraw from the study at any time without facing any consequences. In line with the principles of the Declaration of Helsinki, the study was conducted in a manner that respected participants, did not harm, and kept participant data strictly confidential.

This study focused on investigating oral hygiene practices and the use of natural compound-based toothpastes (sodium bicarbonate, activated charcoal, and lemon) in relation to dental plaque accumulation and gingival health among young adults from both urban and rural settings. A minimum sample size of 101 participants was required, with the effect size determined based on findings from previous studies [12,13,14,15].

This cross-sectional study included 101 young adults from Romania, recruited at the Clinic of the West University “Vasile Goldiș” in Arad. Participants sought routine dental care and provided consent to undergo a clinical evaluation for the study. Each participant completed a structured questionnaire regarding oral hygiene habits, followed by a clinical examination that included plaque disclosure, photographic documentation, and assessment of gingival and plaque indices.

### 2.1. Procedure Methodology

A total of 101 young adults, aged between 20 and 41 years, were recruited from the “Vlaicu” Polyclinic of the West University “Vasile Goldiș” in Arad, Romania. Participants were informed about the purpose of the study, and written consent was then obtained in accordance with the approved ethical procedures.

**Recruitment and Eligibility**

All potential participants underwent initial screening to ensure compliance with the inclusion and exclusion criteria.

**The following were the conditions for inclusion:**

- Between the ages of 20 and 41;
- Live in either the city or the country in Arad County;
- In excellent health overall, with no heart disease, diabetes, or other diseases that could affect oral health;
- With at least 20 natural teeth and no extensive prosthetic rehabilitation, to ensure reliable and comparable plaque and gingival index assessment;
- Have received no orthodontic or periodontal therapy within the past 12 months, as recent treatment may alter gingival indices and plaque accumulation [16];
- Willing to follow study instructions, including using their self-reported habitual toothpaste formulation during the supervised brushing session and applying the standard Bass toothbrushing technique;
- Provide written consent before taking part.

**Criteria for exclusion included:**

- People who have known allergies or are very sensitive to any of the natural toothpaste ingredients that were tested (sodium bicarbonate, activated charcoal, lemon) or the plaque-revealing agent that was used in the clinical study;
- Individuals who have used antibiotics, anti-inflammatories, or antibacterial mouthwashes within the last four weeks were excluded, as such agents can transiently alter the oral microbiota and gingival inflammation [17];
- Women who are pregnant or breastfeeding, because hormones can change the health of the gums;
- People who are currently getting braces, removable prostheses, or extensive fixed prosthetic restorations that could make it challenging to examine for plaque;
- People who have had craniofacial abnormalities, cleft lip/palate, or maxillofacial surgical interventions (other than minor oral surgery like extractions);
- People who have active cavities that need urgent restorative treatment or acute oral infections at the time of the examination;
- Individuals who were unable or unwilling to undergo both pre- and post-brushing evaluations were excluded.

**Questionnaire**

Before the clinical examination, all 101 participants completed a structured questionnaire documenting oral-hygiene practices, including frequency of toothbrushing (once, twice, or three times daily, or after meals), type of toothbrush used (manual or electric), and the use of adjunctive cleaning methods such as dental floss, interdental stimulators, and mouthrinse. Additional items addressed lifestyle factors (dietary habits, smoking status) and self-reported dental visits.

Group Allocation and Toothpaste Use

Participants were categorised into three groups according to their self-reported habitual use of natural toothpaste formulations, which were then used for the supervised brushing session:

Sodium bicarbonate group (*n* = 42).

Activated charcoal group (*n* = 27).

Lemon-based group (*n* = 32).

Group sizes differed according to the prevalence of each toothpaste type reported by participants. No random assignment was performed, as the study was designed to observe natural behavioural and product-use patterns rather than to test product efficacy under controlled trial conditions.

All dentifrices were commercially available products containing the respective natural ingredients as the main active component: sodium bicarbonate toothpaste (Parodontax™ Baking Soda, Haleon/GlaxoSmithKline, Dungarvan, Ireland), activated charcoal toothpaste (Signal™ White Now Charcoal, Unilever, Rotterdam, Netherlands), and lemon-based toothpaste (EcoDenta™ Citrus Whitening, BIOK Laboratorija, Klevine, Lithuania).

**Brushing Protocol**

To ensure standardisation, participants were instructed to perform toothbrushing under supervision using the Bass technique, widely recognised as an effective method for removing plaque along the gingival margin and interproximal areas [18,19]. All brushing sessions were conducted between 9:00 a.m. and 12:00 p.m., at least 2 h after participants’ last meal or drink, to standardise conditions and minimise variability related to circadian changes or recent food intake. Participants confirmed that they had not brushed their teeth earlier that morning before the session. A calibrated examiner demonstrated the technique before participants brushed independently for a standardised period of 2 min. All participants used soft-bristled toothbrushes provided by the investigators to minimise variability due to differences in brush design.

**Clinical Evaluation**

Clinical examination was performed both before brushing (baseline) and immediately after brushing. This immediate post-brushing evaluation was intentionally designed to capture short-term clinical effects of a single standardised brushing session, in line with the study’s objective to assess immediate plaque and gingival changes rather than long-term outcomes. The following indices were recorded:

The Silness–Löe Plaque Index [20] is used to assess plaque accumulation at the gingival margin of selected index teeth. A plaque disclosing agent was applied to facilitate visualisation of deposits.

The Löe–Silness Gingival Index modified by Lobene [21] is used to evaluate gingival inflammation, including changes in colour, contour, and the presence of bleeding on probing.

In addition to plaque and gingival indices, examiners recorded other oral health parameters, including the presence of carious lesions, dental restorations, gingival recessions, and spontaneous gingival bleeding. Standardised intraoral photographs were taken at baseline and post-brushing to document outcomes visually.

**Examiner Calibration and Data Collection**

All clinical examinations were performed by two calibrated investigators (dentists with experience in periodontal assessment) who were specifically trained in applying the Silness–Löe Plaque Index and the Löe–Silness Gingival Index (modified by Lobene) to ensure inter-examiner reliability. Calibration was established through repeated assessments on a pilot group (*n* = 10) before data collection, yielding a Cohen’s kappa coefficient of 0.87, indicating strong agreement between examiners. Examinations were performed under standardised lighting conditions in dental chairs equipped with overhead lights.

Clinical data were recorded on individual case report forms and subsequently transferred into a digital database. Double data entry was used to minimise transcription errors. Statistical analysis was performed after the data were cleaned and verified.

#### Statistical Analysis

All analyses were conducted in R (version 4.4.x) within RStudio (2025.05.1+513.pro3, Posit, Boston, MA, USA). Prior to analysis, the data were screened for range errors and internal consistency; analyses were conducted using complete cases with no imputation. Categorical variables are reported as *n*; approximately continuous variables (e.g., age) as mean (SD) if normally distributed and median (IQR) otherwise. Two-sided tests were used throughout with α = 0.05. Where expected cell counts were <5, Fisher’s exact test replaced Pearson’s χ^2^. Effect sizes accompany *p*-values whenever appropriate: Cramér’s V for χ^2^ tests, Cohen’s d for standardised mean differences, and rank-biserial r for Wilcoxon contrasts; all effect sizes are presented with 95% confidence intervals. Distributional assumptions for continuous variables were examined with Shapiro–Wilk tests and visual diagnostics (histograms and Q–Q plots). Between urban vs. rural groups, age was compared with independent-samples *t*-tests when normality and variance homogeneity (Levene’s test) were supported; otherwise, the Mann–Whitney U test was used. For categorical behaviours and clinical categories (e.g., brushing frequency; floss/interdental/mouthrinse use; presence/number of dental lesions; periodontal status), Pearson’s χ^2^ tests were used to compare groups. Because clinical indices were recorded before and after a standardised brushing session on the same participants, two classes of comparisons were prespecified. First, between-group differences (by toothpaste group and by residence) were assessed separately at each time point using Pearson’s χ^2^ (or Fisher’s exact). Results are reported as contrasts of *n* at each time point; *p*-values shown in the summary tables correspond to the between-group comparisons at that time point. To investigate relations between clinical indices and toothpaste groups beyond bivariate tables, we fit a multinomial logistic regression with the toothpaste adjunct group (reference: bicarbonate) as the outcome and pre-/post-brushing plaque and gingival categories as predictors, adjusting for age and residence (urban/rural). Models were estimated using maximum likelihood (e.g., nnet::multinom). Standard errors were based on the observed information matrix, and odds ratios (ORs) with 95% CIs are reported. Because several cells were sparse or empty (e.g., categories absent in a group), we prospectively screened for quasi-/complete separation (e.g., brglm2::detect_separation). Where separation was detected, we report the conventional MLEs for completeness and conducted a bias-reduced (Firth-type) sensitivity analysis (brglm2::brmultinom) to verify direction and significance of effects; Inter-examiner reliability for the Silness–Löe Plaque Index and the Löe–Silness Gingival Index (modified by Lobene) was quantified using the intraclass correlation coefficient (ICC), two-way mixed effects, absolute agreement, single rater, with 95% CIs (e.g., irr::icc). Agreement benchmarks followed conventional thresholds. Graphical summaries (bar charts and paired before/after displays) were produced with ggplot2; results tables were generated with gtsummary/broom to ensure consistent formatting. Given the study’s exploratory, cross-sectional design, no multiplicity adjustment was applied to families of secondary comparisons; primary inferences concern the distributions of plaque and gingival indices across groups at each time point and the direction/robustness of associations in the multinomial model.

## 3. Results

A total of 101 participants were included in the study, with a mean age of 23.32 years (SD = 5.26). The majority resided in urban areas, accounting for 70 individuals (69.3%).

Participants were categorised by their self-reported habitual toothpaste (baking soda, charcoal, or lemon); group sizes reflect the prevalence of each product in the sample.

Most participants reported brushing their teeth twice daily (56.4%); a minority brushed them once daily (5.0%). See Table 1 for full distributions. Dental floss was not used by 42 participants (41.6%), and interdental stimulators were not used by 65 (64.4%). Similarly, 26 participants (25.7%) reported not using mouthwash.

Concerning oral health status, 42 participants (41.6%) reported the absence of carious lesions or restorations. Among those with lesions, 37 (36.6%) had between one and two, and 22 (21.8%) had between three and four lesions. Regarding periodontal status, 73 participants (72.3%) reported no signs of gingival inflammation, while 24 (23.8%) experienced simple gingival bleeding, and 4 (4.0%) presented with gingival recessions.

Clinical indices revealed that, prior to brushing, the Silness–Loe plaque index indicated a thin adherent film at the gingival margin and adjacent tooth surfaces in 79 participants (78.2%). The gingival index of Loe and Silness, modified by Lobene, showed that 73 participants (72.3%) had normal gingiva, 23 (22.8%) displayed mild inflammation, and 5 (5.0%) presented with moderate inflammation.

Regarding the type of toothpaste used, 42 participants (41.6%) reported using baking soda-based products, 27 (26.7%) used charcoal-based products, and 32 (31.7%) used lemon-based products. After brushing, gingival health improved, with 78 participants (77.2%) demonstrating normal gingiva, 18 (17.8%) showing mild inflammation, and 5 (5.0%) remaining with moderate inflammation. The Silness–Loe plaque index after brushing indicated the persistence of a thin adherent film in 23 participants (22.8%) (Table 1).

**Figure 1:**
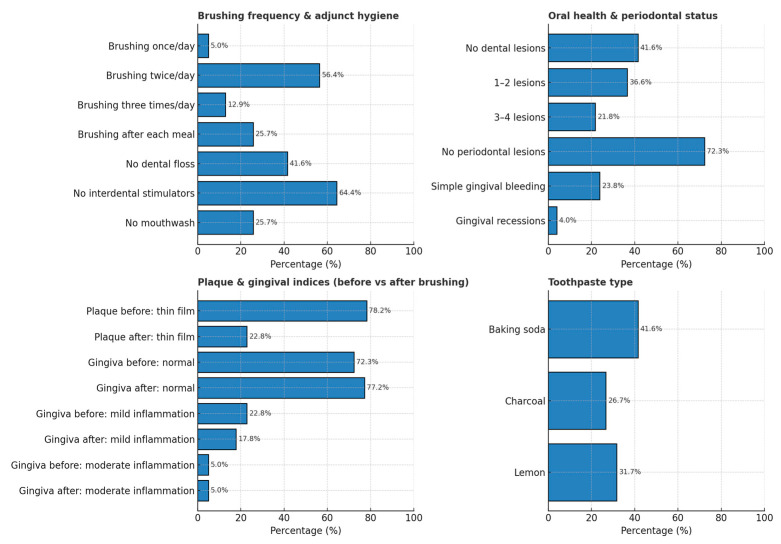
Oral Hygiene Practices, Clinical Indices, and Toothpaste Use Among Study Participants.

Figure 1 illustrates the distributions of oral-hygiene practices, oral-health status, plaque/gingival indices (before vs. after brushing), and habitual toothpaste type among study participants: Brushing frequency and adjunct hygiene: most participants brushed twice daily (56.4%).

Oral health and periodontal status: no carious lesions/restorations in 41.6%; no periodontal inflammation in 72.3%.

Plaque and gingival indices: thin adherent plaque decreased from 78.2% before brushing to 22.8% after; normal gingiva increased from 72.3% to 77.2%.

Toothpaste type: baking soda–based products were most common (41.6%), followed by lemon (31.7%) and charcoal (26.7%).

101 participants were included, comprising 31 from rural settings and 70 from urban areas. The mean age was 23.9 years (SD = 6.9) in the rural group and 23.0 years (SD = 4.4) in the urban group, with no significant difference between groups (*p* = 0.434). Age ranges were similar, spanning from 20 to 41 years in rural participants and from 20 to 36 years in urban participants.

Brushing frequency differed markedly between groups (*p* < 0.001). In the rural cohort, 5 participants (16.1%) brushed once daily, 9 (29.0%) brushed twice daily, 4 (12.9%) brushed three times daily, and 13 (41.9%) brushed after each meal. In contrast, no urban participant reported brushing once daily, while 48 (68.6%) brushed twice daily, 9 (12.9%) three times daily, and 13 (18.6%) after each meal. Thus, urban participants were significantly more likely to report brushing twice daily, whereas rural participants predominantly reported brushing after each meal. The use of dental floss did not differ significantly between groups (*p* = 0.206). Among rural respondents, 21 (67.7%) reported using dental floss, compared to 38 (54.3%) in the urban group. Similarly, interdental stimulators were used by 13 (41.9%) rural participants and 23 (32.9%) urban participants, with no statistical significance observed (*p* = 0.380). The use of mouthwash also showed no significant variation between groups (*p* = 0.615), with 22 (71.0%) rural participants and 53 (75.7%) urban participants reporting its use.

The prevalence of dental lesions or restorations was broadly comparable across settings, with 17 (54.8%) rural and 42 (60.0%) urban participants reporting lesions (*p* = 0.627). When stratified by lesion count, 8 (25.8%) rural and 29 (41.4%) urban respondents reported one to two lesions, 9 (29.0%) and 13 (18.6%) reported three to four lesions, and 14 (45.2%) and 28 (40.0%) were free of lesions, respectively; these differences were not statistically significant (*p* = 0.266).

Periodontal status, however, demonstrated a significant divergence between groups (*p* = 0.002). In the rural cohort, 17 (54.8%) were free of periodontal lesions, 14 (45.2%) presented with simple bleeding lesions, and none exhibited gingival recessions. Conversely, among urban participants, 56 (80.0%) were lesion-free, 10 (14.3%) reported simple bleeding lesions, and 4 (5.7%) displayed gingival recessions.

Assessment of the Silness–Loe plaque index prior to brushing revealed no significant difference between groups (*p* = 0.360). The majority presented with a thin adherent film detectable with probing, affecting 26 (83.9%) of rural and 53 (75.7%) of urban participants. Gingival status prior to brushing, however, differed significantly (*p* = 0.002). In the rural group, 18 (58.1%) had normal gingiva, 8 (25.8%) showed mild inflammation, and 5 (16.1%) moderate inflammation. In contrast, 55 (78.6%) of urban participants had normal gingiva, 15 (21.4%) had mild inflammation, and none had moderate inflammation.

Regarding toothpaste selection, a highly significant variation emerged (*p* < 0.001). Baking soda-based products were reported by 8 (25.8%) rural and 34 (48.6%) urban participants, charcoal-based products by 18 (58.1%) and 9 (12.9%), and lemon-based products by 5 (16.1%) and 27 (38.6%), respectively. This indicates a preference for charcoal among rural participants, while baking soda and lemon products were more frequently reported in the urban group (Table 2).

The analysis included 101 participants, divided into Group 0 (N = 78; baseline Silness–Löe plaque index 0, absence of plaque) and Group 1 (N = 23; baseline Silness–Löe plaque index 1, adherent pellicle). Pearson’s χ^2^ test was applied to categorical variables, and a linear model ANOVA to age.

For the Löe & Silness gingival index (Lobene) after brushing, group distributions differed markedly (*p* < 0.001). Normal gingiva was observed in 74 (94.9%) in Group 0 versus 4 (17.4%) in Group 1, while mild inflammation occurred in 4 (5.1%) versus 14 (60.9%), and moderate inflammation in 0 (0.0%) versus 5 (21.7%). These results indicate a substantially more favourable post-brushing gingival status in Group 0.

The type of toothpaste varied significantly between groups (*p* < 0.001). Bicarbonate use was reported by 34 (43.6%) in Group 0 and 8 (34.8%) in Group 1; charcoal by 27 (34.6%) in Group 0 and 0 (0.0%) in Group 1; and lemon by 17 (21.8%) in Group 0 and 15 (65.2%) in Group 1. The predominance of lemon and the absence of charcoal in Group 1 reflect distinct oral-hygiene choices relative to Group 0.

For the Löe & Silness gingival index (Lobene) before brushing, differences were also significant (*p* < 0.001). Normal gingiva was present in 69 (88.5%) in Group 0 and 4 (17.4%) in Group 1; mild inflammation in 9 (11.5%) versus 14 (60.9%); and moderate inflammation in 0 (0.0%) versus 5 (21.7%). Group 1 exhibited greater baseline gingival inflammation.

For the Silness–Löe plaque index before brushing, the distribution favoured Group 0 (*p* = 0.004). Absence of plaque was recorded in 22 (28.2%) in Group 0 and 0 (0.0%) in Group 1, whereas an adherent pellicle was present in 56 (71.8%) and 23 (100.0%), respectively. Thus, all participants in Group 1 had detectable plaque at baseline.

The presence of signs of gingival inflammation did not differ significantly (*p* = 0.097). No lesions were noted in 59 (75.6%) in Group 0 and 14 (60.9%) in Group 1; simple bleeding lesions in 15 (19.2%) versus 9 (39.1%); and gingival recessions in 4 (5.1%) versus 0 (0.0%). The pattern suggests a higher burden in Group 1, but the difference did not reach statistical significance.

Carious lesion/restorations by category differed significantly (*p* < 0.001). One to two lesions occurred in 24 (30.8%) in Group 0 and 13 (56.5%) in Group 1; three to four lesions in 12 (15.4%) versus 10 (43.5%); and no lesions in 42 (53.8%) versus 0 (0.0%). The binary summary corroborated this finding, with any lesion/restoration present in 36 (46.2%) in Group 0 and 23 (100.0%) in Group 1 (*p* < 0.001).

Mouthwash use was not significantly different (*p* = 0.095), reported by 61 (78.2%) in Group 0 and 14 (60.9%) in Group 1, while 17 (21.8%) and 9 (39.1%) reported non-use. Use of interdental stimulators was likewise non-significant (*p* = 0.372), reported by 26 (33.3%) in Group 0 and 10 (43.5%) in Group 1, with non-use in 52 (66.7%) and 13 (56.5%).

Dental floss use was significantly more frequent in Group 0 (*p* = 0.033), reported by 50 (64.1%) versus 9 (39.1%), while non-use occurred in 28 (35.9%) versus 14 (60.9%). This suggests more favourable interdental hygiene in Group 0.

Brushing frequency differed significantly (*p* < 0.001). Once-daily brushing was reported by 0 (0.0%) in Group 0 and 5 (21.7%) in Group 1; twice-daily by 44 (56.4%) and 13 (56.5%); three times daily by 13 (16.7%) and 0 (0.0%); and after each meal by 21 (26.9%) and 5 (21.7%). Group 1 had a higher proportion of individuals who brushed once daily and no instances of those who brushed three times daily.

Residence did not differ significantly (*p* = 0.318), with rural residence in 22 (28.2%) in Group 0 and 9 (39.1%) in Group 1, and urban residence in 56 (71.8%) and 14 (60.9%). Age was also non-significant by ANOVA (*p* = 0.454), with mean (SD) values of 23.1 (4.9) years in Group 0 and 24.0 (6.5) years in Group 1; the ranges were 20.0–41.0 and 20.0–36.0, respectively (Table 3).

Distributions across toothpaste groups—Bicarbonate (N = 42), Charcoal (N = 27), and Lemon (N = 32)—differed significantly both before and after brushing for the Silness–Löe Plaque Index and the Löe & Silness Gingival Index (modified) (Pearson’s χ^2^, *p* < 0.001 at each time point), indicating non-random heterogeneity among groups. For the Silness–Löe Plaque Index, absence of plaque increased after brushing in all groups, with the largest absolute gain in the Charcoal group. Before brushing, the absence of plaque was observed in Bicarbonate 17 (40.5%), Charcoal 5 (18.5%), and Lemon 0 (0.0%), contributing to a Total 22 (21.8%). After brushing, the absence of plaque rose to 34 (81.0%) for Bicarbonate, 27 (100.0%) for Charcoal, and 17 (53.1%) for Lemon, with a total of 78 (77.2%). Complementarily, Grade I decreased from Bicarbonate 25 (59.5%), Charcoal 22 (81.5%), and Lemon 32 (100.0%) to Bicarbonate 8 (19.0%), Charcoal 0 (0.0%), and Lemon 15 (46.9%), with the Total declining from 79 (78.2%) to 23 (22.8%). These patterns reflect a substantial immediate reduction in detectable plaque following a standardised brushing session, most pronounced in Charcoal, for which the absence of plaque increased by 22 individuals (from 5 (18.5%) to 27 (100.0%)). The reported *p*-values pertain to between-group comparisons at each time point; within-group changes before and after are described descriptively. For the Löe & Silness Gingival Index (modified), group differences were also significant before and after brushing (*p* < 0.001), although the short-interval change was modest. Before brushing, normal gingiva was present in Bicarbonate 29 (69.0%), Charcoal 27 (100.0%), and Lemon 17 (53.1%), totalling 73 (72.3%). After brushing, normal gingiva was observed in Bicarbonate 34 (81.0%), Charcoal 27 (100.0%), and Lemon 17 (53.1%), totalling 78 (77.2%) cases. Mild inflammation decreased in bicarbonate from 13 (31.0%) to 8 (19.0%) and remained unchanged in lemon at 10 (31.2%), while charcoal showed 0 (0.0%) at both time points; moderate inflammation was confined to lemon at 5 (15.6%) before and after brushing, with a total of 5 (5.0%) unchanged. Overall, the gingival findings suggest a ceiling effect in Charcoal, a small improvement in Bicarbonate, and no detectable short-term change in Lemon, consistent with limited inflammatory shifts over a single brushing session (Table 4).

## 4. Discussion

In this exploratory cross-sectional cohort of 101 young adults, a single supervised Bass brushing session using natural dentifrices was associated with short-term improvements in plaque control and gingival status. Due to the study design, the findings should be interpreted strictly as associations observed within a single time point comparison, rather than indications of product efficacy. The post-brushing improvements—such as the increase in the proportion of participants presenting with a normal gingival appearance and the decrease in visible adherent pellicle—are consistent with previously documented short-term outcomes of supervised toothbrushing sessions, regardless of the dentifrice used [14,18]. Similar short-term benefits in plaque control have also been demonstrated with natural oral care products such as herbal or plant-based mouthrinses, which in some cases achieved comparable efficacy to chlorhexidine when used adjunctively [22]. As this was an observational cross-sectional study, the results reflect short-term associations rather than causal effects.

The immediate before–after assessment was intentionally selected to quantify short-term brushing outcomes directly relevant to daily oral-hygiene behaviours and clinical instruction.

Oral hygiene behaviours varied considerably across participants. More than half brushed twice daily, yet only 58.4% reported flossing, and fewer than 40% used interdental stimulators. This underuse of interdental aids aligns with findings from other studies in young adults, which consistently demonstrate lower adherence to interdental cleaning despite its established importance in preventing gingival inflammation [13,15]. Our correlation analyses confirmed this relationship, showing that the use of adjunctive cleaning methods was inversely associated with post-brushing gingival inflammation (ρ = −0.36), consistent with the Cochrane review which indicates that adding floss or interdental brushes to toothbrushing reduces gingivitis and plaque, with interdental brushes often showing greater efficacy [23]. Similarly, a recent meta-analysis confirmed that two minutes of brushing removes significantly more plaque than one minute, regardless of brush type [24].

Toothpaste selection was associated with different patterns of clinical indices. Sodium bicarbonate–based formulations were more common among participants with favourable gingival responses, whereas lemon-based formulations predominated among those with higher levels of inflammation. The observed gingival improvements with bicarbonate formulations are biologically plausible, as systematic reviews have shown that single-use brushing with bicarbonate formulations results in superior plaque removal compared to conventional pastes. However, long-term outcomes remain variable [25]. In contrast, charcoal-based dentifrices have not consistently demonstrated clinical benefits and are associated with potential safety concerns—particularly abrasivity and lack of fluoride [26]—which is consistent with our observation of limited gingival improvement in this group. Lemon-based formulations were associated with moderate gingival benefits, but due to their acidity, they require buffering and fluoride inclusion to prevent potential enamel erosion.

Urban–rural differences were also evident. Urban participants reported brushing twice daily more frequently and had fewer bleeding lesions, while rural participants brushed more often after meals but exhibited higher rates of gingival bleeding. These disparities likely reflect sociocultural and behavioural influences on oral health, consistent with prior European population-based studies [13]. Similar patterns of variability in clinical practice behaviours and material selection have been reported in Romanian dental settings, highlighting the influence of practitioner education and resource availability on treatment outcomes [27]. Given that the European Region reports the highest global prevalence of oral diseases, these findings underscore the public health relevance of promoting proper oral hygiene behaviours in both urban and rural communities [28,29].

Subgroup analysis based on baseline plaque status provided additional insights. Participants without visible plaque (Group 0) demonstrated markedly better gingival outcomes after brushing, with 94.9% exhibiting normal gingiva compared with only 17.4% among those with adherent pellicle (Group 1). Group 1 participants also exhibited higher rates of carious lesions and less frequent brushing and flossing habits. These results underscore the importance of consistent toothbrushing frequency and interdental cleaning, in line with prior clinical evidence [13,14].

From a clinical perspective, these findings support a preventive strategy emphasising standardised brushing instruction, twice-daily brushing, and regular interdental cleaning, combined with dentifrice choices that balance natural appeal with safety and fluoride content. As highlighted by professional guidelines, toothpastes bearing the ADA Seal must contain fluoride, and whitening effects typically target extrinsic stains rather than intrinsic enamel colour [30].

The subgroup distributions across toothpaste groups confirmed non-random trends. Charcoal formulations showed the largest observed pre–post difference in plaque scores, yet this did not translate into significant gingival improvements, likely due to their abrasivity [25]. Sodium bicarbonate was associated with more favourable plaque and gingival indices, supporting previous evidence of its effectiveness in biofilm disruption and gingivitis reduction [25]. Nonetheless, the present findings are exploratory and do not establish comparative product efficacy Lemon-based products, however, were linked with persistent mild-to-moderate inflammation, consistent with the erosive potential of acidic formulations when unbuffered [9]. These group differences should be viewed as descriptive patterns that may guide future hypothesis-driven research. The study design does not allow conclusions regarding the comparative effectiveness of the dentifrices.

Previous laboratory and profilometric investigations have shown that charcoal-based toothpastes are more abrasive on enamel and dentin surfaces than non-charcoal formulations, warranting caution in their frequent use [31,32,33,34]. The modest gingival gains observed in this group may therefore reflect a trade-off between enhanced cleaning and potential micro-abrasion, possibly influenced by participants’ cautious brushing behaviour.

Emerging alternatives—such as probiotic or enzyme-enriched dentifrices—may offer additional benefits by modulating the oral microbiota and biofilm activity, complementing mechanical plaque control [35,36].

The current findings underscore the importance of individualised oral hygiene counselling, particularly among young adults in rural settings, who demonstrate higher gingival bleeding rates despite frequent self-reported brushing. Even brief, supervised “micro-sessions” of Bass brushing can improve clinical indices and serve as effective motivational tools in routine dental care. Clinicians should recommend fluoride-containing sodium bicarbonate formulations for patients seeking “natural” products while discouraging charcoal-based pastes unless the safety of these products (abrasivity, fluoride content) is well established. Given the underuse of interdental aids, personalised demonstrations of proper technique could further enhance outcomes. At the population level, targeted education programs are needed to bridge urban–rural disparities and promote equitable access to safe, evidence-based hygiene products and practices.

Recent studies in periodontology also emphasise that consistent plaque control combined with evidence-based adjunctive therapies is essential for maintaining long-term periodontal stability [37,38,39,40,41].

Because this was a single brushing session, the observed differences should be interpreted as indicative trends rather than proof of sustained efficacy.

In summary, consistent toothbrushing frequency, correct technique, and the use of safe natural dentifrices can improve short-term gingival outcomes in young adults. While measurable benefits were observed after a single supervised session, the findings underscore the importance of maintaining sustained daily hygiene routines to prevent biofilm accumulation and gingival inflammation. The differential effects of bicarbonate-, lemon-, and charcoal-based products confirm that toothpaste selection is not neutral; natural dentifrices must be evaluated not only for cosmetic appeal but also for fluoride content, abrasivity, and clinical performance. Future multicentre, randomised, longitudinal studies are warranted to validate these short-term findings and assess long-term safety. Integrating personalised counselling with public health strategies remains vital for reducing the burden of gingival disease among young adults.

## 5. Study Limitations and Future Perspectives

This study has several limitations that should be considered when interpreting the findings. First, the sample size was relatively small, comprising only 101 participants from a single university clinic in Arad. Therefore, the results cannot be generalised to all young adults in Romania or other populations. To improve external validity, future studies should include larger, multicentre samples and, ideally, participants from different regions or countries.

Second, the cross-sectional design evaluated only immediate pre- and post-brushing outcomes and did not assess the long-term effects of natural dentifrices on plaque control, gingival health, or enamel integrity. Longitudinal studies with extended follow-up periods are therefore required to determine the durability of these effects.

Third, although all participants were instructed in the Bass brushing technique, inter-individual differences in manual dexterity and brushing skill may have influenced outcomes. Future studies could minimise this variability by employing standardised brushing training or digital brushing monitors.

Fourth, reliance on self-reported questionnaire data introduces potential recall and social desirability biases, particularly regarding the frequency of interdental cleaning. Additionally, only a limited range of natural toothpastes—sodium bicarbonate, activated charcoal, and lemon-based formulations—were evaluated. These were selected due to their market availability and popularity; however, other natural ingredients such as turmeric, coconut oil, and herbal extracts, also warrant investigation. Future research should compare a wider range of natural formulations using randomised controlled designs.

Despite these limitations, the present study provides valuable insights into oral-hygiene behaviours among young adults and the potential short-term effects of natural dentifrices on plaque control and gingival health. Future research should aim to develop safe, standardised natural formulations, enhance oral health education across urban and rural settings, and assess the cost-effectiveness of integrating natural products into preventive dental care. Moreover, the relatively small sample size may have limited statistical power and increased the risk of type II errors. Consequently, these findings should be interpreted with caution and validated through larger, multicentre investigations involving more diverse populations.

## 6. Conclusions

This cross-sectional exploratory study documented short-term changes in plaque and gingival indices following a single supervised Bass brushing session among young adults. As expected, mechanical plaque removal occurred across all groups; however, standardised supervision allowed us to characterise immediate pre–post differences and to identify behavioural factors associated with gingival responses. Brushing frequency and the use of interdental aids were associated with more favourable post-brushing indices, highlighting the importance of maintaining consistent daily oral hygiene routines.

Although all dentifrice groups exhibited short-term plaque reduction, participants habitually using sodium bicarbonate and lemon formulations appeared to show slightly more favourable gingival responses than those using charcoal-based products. These observations should be regarded as preliminary, descriptive, and non-causal, and they do not imply comparative efficacy of the products.

Urban–rural differences in hygiene behaviours and gingival status highlight the need for targeted oral health education, particularly in rural communities. Future randomised, longitudinal studies with controlled product allocation are necessary to determine whether the short-term associations observed here persist over time and to clarify the long-term safety and clinical relevance of natural dentifrice formulations. These results are preliminary and hypothesis-generating.

## Figures and Tables

**Table 1:** Demographic characteristics, oral hygiene practices, toothpaste type, and clinical indices of the study participants.

Variable	Category	*n* (%)/Mean (SD)
**Sample size**		101
**Age (years)**	Mean (SD)	23.32 (5.26)
**Environment**	Urban	70 (69.3)
**Daily brushing frequency**	Once a day	5 (5.0)
	Twice a day	57 (56.4)
	Three times a day	13 (12.9)
	After each meal	26 (25.7)
**Use of dental floss**	No	42 (41.6)
**Use of interdental stimulators**	No	65 (64.4)
**Use of mouthwash**	No	26 (25.7)
**Presence of carious lesions/fillings**	No	42 (41.6)
**Number of carious lesions/fillings**	1–2	37 (36.6)
	3–4	22 (21.8)
	None	42 (41.6)
**Presence of signs of gingival inflammation**	None	73 (72.3)
	Gingival recessions	4 (4.0)
	Simple (gingival bleeding)	24 (23.8)
**Silness-Loe plaque index before brushing**	Grade 1 = thin film adherent at gingival margin and adjacent tooth surfaces, detectable with probe	79 (78.2)
**Loe & Silness gingival index modified by Lobene before brushing**	Normal gingiva	73 (72.3)
	Mild inflammation	23 (22.8)
	Moderate inflammation	5 (5.0)
**Toothpaste used**	Baking soda	42 (41.6)
	Charcoal	27 (26.7)
	Lemon	32 (31.7)
**Loe & Silness gingival index modified by Lobene after brushing**	Normal gingiva	78 (77.2)
	Mild inflammation	18 (17.8)
	Moderate inflammation	5 (5.0)
**Silness-Loe plaque index after brushing**	Thin film adherent at gingival margin and adjacent tooth surfaces, detectable with probe	23 (22.8)

**Table 2:** Comparison of demographic characteristics, oral hygiene.

Variable	Category	Rural (N = 31)	Urban (N = 70)	Total (N = 101)	*p*-Value
Age (years)	Mean (SD)	23.9 (6.9)	23.0 (4.4)	23.3 (5.3)	0.434 ^1^
	Range	20.0–41.0	20.0–36.0	20.0–41.0	
Daily brushing frequency	Once a day	5 (16.1%)	0 (0.0%)	5 (5.0%)	<0.001 ^2^
	Twice a day	9 (29.0%)	48 (68.6%)	57 (56.4%)	
	Three times a day	4 (12.9%)	9 (12.9%)	13 (12.9%)	
	After each meal	13 (41.9%)	13 (18.6%)	26 (25.7%)	
Use of dental floss	Yes	21 (67.7%)	38 (54.3%)	59 (58.4%)	0.206 ^2^
	No	10 (32.3%)	32 (45.7%)	42 (41.6%)	
Use of interdental stimulators	Yes	13 (41.9%)	23 (32.9%)	36 (35.6%)	0.380 ^2^
	No	18 (58.1%)	47 (67.1%)	65 (64.4%)	
Use of mouthwash	Yes	22 (71.0%)	53 (75.7%)	75 (74.3%)	0.615 ^2^
	No	9 (29.0%)	17 (24.3%)	26 (25.7%)	
Presence of carious lesions/fillings	Yes	17 (54.8%)	42 (60.0%)	59 (58.4%)	0.627 ^2^
	No	14 (45.2%)	28 (40.0%)	42 (41.6%)	
Number of carious lesions/fillings	1–2	8 (25.8%)	29 (41.4%)	37 (36.6%)	0.266 ^2^
	3–4	9 (29.0%)	13 (18.6%)	22 (21.8%)	
	None	14 (45.2%)	28 (40.0%)	42 (41.6%)	
Presence of signs of gingival inflammation	None	17 (54.8%)	56 (80.0%)	73 (72.3%)	0.002 ^2^
	Gingival recessions	0 (0.0%)	4 (5.7%)	4 (4.0%)	
	Simple (gingival bleeding)	14 (45.2%)	10 (14.3%)	24 (23.8%)	
Silness–Loe plaque index before brushing	No plaque	5 (16.1%)	17 (24.3%)	22 (21.8%)	0.360 ^2^
	Thin film adherent at gingival margin and adjacent surfaces	26 (83.9%)	53 (75.7%)	79 (78.2%)	
Loe & Silness gingival index (modified by Lobene) before brushing	Normal gingiva	18 (58.1%)	55 (78.6%)	73 (72.3%)	0.002 ^2^
	Mild inflammation	8 (25.8%)	15 (21.4%)	23 (22.8%)	
	Moderate inflammation	5 (16.1%)	0 (0.0%)	5 (5.0%)	
Toothpaste used	Baking soda	8 (25.8%)	34 (48.6%)	42 (41.6%)	<0.001 ^2^
	Charcoal	18 (58.1%)	9 (12.9%)	27 (26.7%)	
	Lemon	5 (16.1%)	27 (38.6%)	32 (31.7%)	
Loe & Silness gingival index (modified by Lobene) after brushing	Normal gingiva	18 (58.1%)	60 (85.7%)	78 (77.2%)	<0.001 ^2^
	Mild inflammation	8 (25.8%)	10 (14.3%)	18 (17.8%)	
	Moderate inflammation	5 (16.1%)	0 (0.0%)	5 (5.0%)	
Silness–Loe plaque index after brushing	No plaque	22 (71.0%)	56 (80.0%)	78 (77.2%)	0.318 ^2^
	Thin film adherent at gingival margin and adjacent surfaces	9 (29.0%)	14 (20.0%)	23 (22.8%)	

^1^ *p*-values calculated using the independent-samples *t*-test; ^2^ *p*-values calculated using Pearson’s χ^2^ test.

**Table 3:** Comparison of Oral and Gingival Health Parameters Between Participants With and Without Baseline Plaque (N = 101).

Domain/Variable	Category	Absence of Plaque (N = 78)	Baseline Silness–Löe Plaque Index (N = 23)	Total (N = 101)	*p* Value	Test
Löe & Silness gingival index (Lobene)—after brushing	normal gingiva	74 (94.9%)	4 (17.4%)	78 (77.2%)	<0.001	Pearson’s χ^2^
	mild inflammation	4 (5.1%)	14 (60.9%)	18 (17.8%)		
	moderate inflammation	0 (0.0%)	5 (21.7%)	5 (5.0%)		
Toothpaste used	Bicarbonate	34 (43.6%)	8 (34.8%)	42 (41.6%)	<0.001	Pearson’s χ^2^
	Charcoal	27 (34.6%)	0 (0.0%)	27 (26.7%)		
	Lemon	17 (21.8%)	15 (65.2%)	32 (31.7%)		
Löe & Silness gingival index (Lobene)—before brushing	normal gingiva	69 (88.5%)	4 (17.4%)	73 (72.3%)	<0.001	Pearson’s χ^2^
	mild inflammation	9 (11.5%)	14 (60.9%)	23 (22.8%)		
	moderate inflammation	0 (0.0%)	5 (21.7%)	5 (5.0%)		
Silness–Löe plaque index—before brushing	absence of plaque	22 (28.2%)	0 (0.0%)	22 (21.8%)	0.004	Pearson’s χ^2^
	adherent pellicle	56 (71.8%)	23 (100.0%)	79 (78.2%)		
Presence of signs of gingival inflammation	None	59 (75.6%)	14 (60.9%)	73 (72.3%)	0.097	Pearson’s χ^2^
	Gingival recessions	4 (5.1%)	0 (0.0%)	4 (4.0%)		
	Simple (gingival bleeding)	15 (19.2%)	9 (39.1%)	24 (23.8%)		
Carious lesions/restorations (categories)	1–2	24 (30.8%)	13 (56.5%)	37 (36.6%)	<0.001	Pearson’s χ^2^
	3–4	12 (15.4%)	10 (43.5%)	22 (21.8%)		
	None	42 (53.8%)	0 (0.0%)	42 (41.6%)		
Any carious lesions/restoration	Yes	36 (46.2%)	23 (100.0%)	59 (58.4%)	<0.001	Pearson’s χ^2^
	No	42 (53.8%)	0 (0.0%)	42 (41.6%)		
Mouthwash use	Yes	61 (78.2%)	14 (60.9%)	75 (74.3%)	0.095	Pearson’s χ^2^
	No	17 (21.8%)	9 (39.1%)	26 (25.7%)		
Interdental stimulators use	Yes	26 (33.3%)	10 (43.5%)	36 (35.6%)	0.372	Pearson’s χ^2^
	No	52 (66.7%)	13 (56.5%)	65 (64.4%)		
Dental floss use	Yes	50 (64.1%)	9 (39.1%)	59 (58.4%)	0.033	Pearson’s χ^2^
	No	28 (35.9%)	14 (60.9%)	42 (41.6%)		
Brushing frequency (per day)	Once	0 (0.0%)	5 (21.7%)	5 (5.0%)	<0.001	Pearson’s χ^2^
	Twice	44 (56.4%)	13 (56.5%)	57 (56.4%)		
	Three times	13 (16.7%)	0 (0.0%)	13 (12.9%)		
	After each meal	21 (26.9%)	5 (21.7%)	26 (25.7%)		
Residence	Rural	22 (28.2%)	9 (39.1%)	31 (30.7%)	0.318	Pearson’s χ^2^
	Urban	56 (71.8%)	14 (60.9%)	70 (69.3%)		
Age	Mean (SD)	23.1 (4.9)	24.0 (6.5)	23.3 (5.3)	0.454	Linear model ANOVA
	Range	20.0–41.0	20.0–36.0	20.0–41.0		

**Table 4:** Before–after distributions of the Silness–Löe Plaque Index and the Löe & Silness Gingival Index (modified by Lobene) by toothpaste adjunct group (bicarbonate, charcoal, lemon) in young adults (N = 101).

Measure	Category (Score/Definition)	Bicarbonate (Before)	Bicarbonate (After)	Charcoal (Before)	Charcoal (After)	Lemon (Before)	Lemon (After)	Total (Before)	Total (After)	*p* (Before)	*p* (After)
**Silness–Löe Plaque Index**	Absence of plaque	17 (40.5%)	34 (81.0%)	5 (18.5%)	27 (100.0%)	0 (0.0%)	17 (53.1%)	22 (21.8%)	78 (77.2%)	<0.001	<0.001
	Grade I	25 (59.5%)	8 (19.0%)	22 (81.5%)	0 (0.0%)	32 (100.0%)	15 (46.9%)	79 (78.2%)	23 (22.8%)		
**Löe & Silness Gingival Index (modified)**	Normal gingiva	29 (69.0%)	34 (81.0%)	27 (100.0%)	27 (100.0%)	17 (53.1%)	17 (53.1%)	73 (72.3%)	78 (77.2%)	<0.001	<0.001
	Mild inflammation	13 (31.0%)	8 (19.0%)	0 (0.0%)	0 (0.0%)	10 (31.2%)	10 (31.2%)	23 (22.8%)	18 (17.8%)		
	Moderate inflammation	0 (0.0%)	0 (0.0%)	0 (0.0%)	0 (0.0%)	5 (15.6%)	5 (15.6%)	5 (5.0%)	5 (5.0%)		

## Data Availability

All data regarding this manuscript can be requested from the corresponding authors.
